# Open Access: Taking Full Advantage of the Content

**DOI:** 10.1371/journal.pcbi.1000037

**Published:** 2008-03-28

**Authors:** Philip E. Bourne, J. Lynn Fink, Mark Gerstein

**Affiliations:** 1 Skaggs School of Pharmacy and Pharmaceutical Sciences, University of California San Diego, La Jolla, California, United States of America; 2 Program in Computational Biology and Bioinformatics, Department of Molecular Biophysics and Biochemistry, and Department of Computer Science, Yale University, New Haven, Connecticut, United States of America

This Journal and the Public Library of Science (PLoS) at large are standard bearers of the full potential offered through open access publication, but what of you, the reader? For most of you, open access may imply free access to read the journals, but nothing more. There is a far greater potential, but, up to now, little to point to that highlights its tangible benefits. We would argue that, as yet, the full promise of open access has not been realized. There are few persistent applications that collectively use the full on-line corpus, which for the biosciences at least is maintained in PubMed Central (http://www.pubmedcentral.nih.gov/). In short, there are no “killer apps.” Since this readership, beyond any other, would seem to have the ability to change this situation at least in the biosciences, we are issuing a call to action.

While, first and foremost, open access implies downloading and reading full papers for free, additional possibilities exist depending on how the open access material is licensed. PLoS and BioMedCentral (BMC), for example, publish under a Creative Commons Attribution License (CCAL). Under this license authors retain ownership of the copyright for their article, but they allow anyone (commercial or non-commercial) to download, reuse, reprint, modify, distribute, and/or copy articles, as long as the original authors and source are cited. No permission is required from the authors or the publishers. Note that, while this is what PLoS and BMC mean by open access, it is not what other publishers mean, such as the National Academy of Sciences (NAS) in publishing the *Proceedings of the National Academy of Sciences* (*PNAS*) or Oxford University Press (OUP) in publishing the journal *Bioinformatics*. In these two examples, it means free to read, but with variation in what is implied by copyright. For *PNAS*, authors have full rights for print use and readers can freely use figures and tables (with attribution); and for *Bioinformatics*, a Creative Commons license applies, but only for non-commercial use. This issue was recently addressed in more detail in a *PLoS Biology* Editorial [1]. The key point is that these licenses allow us to go far beyond reading material to manipulating it much like data.

Beyond what the licensing laws say about how we might use open access materials, there is then the format in which these materials are available. Papers published as PDFs do not lend themselves to easy manipulation by computer. HTML is better, but the markup has more to do with presentation on a Web page than the semantic content of the paper, which is where the great opportunities lie. XML versions of the paper offer the most promise. When publishers make XML versions available, most conform to the National Library of Medicine (NLM) Document Type Definition (DTD) (http://dtd.nlm.nih.gov). In addition, several markup languages have been developed, such as CellML (http://www.cellml.org) and MathML (http://www.w3.org/Math), which can be used in addition to the NLM DTD to further describe the semantic content of a paper. Semantically aware markup is further elaborated in a systematic fashion in the construction of the semantic Web [2], where the XML tags are related to each other in explicit ontologies. The analogy between an XML file of content offered by a publisher and XML content provided by a database provider should not be missed. As a community, we have been at the forefront of using the latter; will we be at the forefront of using the former? While the DTD and markup languages provide for extensions to meet the needs of each discipline, publishers and researchers have made little use of them to date. This is somewhat of a chicken-and-egg situation. When significant markup is available, it will be used; then again, why go to the trouble of adding significant markup if there are no applications demanding it? The best way out would seem to be to do something significant with the markup we have, which may then inspire authors, publishers, and others to see the research and commercial potential of the corpus.

The use of such markup is a hallmark of Web 2.0 and is manifest in the idea of a mashup. Simply put, a mashup is an integration of Web content from multiple sources to provide a new and more powerful service beyond what can be achieved by any of the individual sources of information it comprises. This type of integration is facilitated if the semantic content from each information source can be identified and thus allow meaningful integration to take place. Specifically in relation to publishing, the mashup manifests the blurring of the distinction between databases and journals, which will continue in future [3],[4].

We already have a significant corpus from a variety of publishers sitting in PubMed Central that is ripe for mashup and other uses. Certainly, the growth rate of the archive hoped for by the NIH has not been met at this time [5], but new laws in the US and elsewhere are changing this situation. Something significant can be done with what we have—so where are the killer apps?

Consider the following applications from our own laboratories. They may not be killer applications, but they begin to illustrate what can be done with this online corpus. The key idea is manipulation of article text as “data” and integration of articles with other bioinformatics information resources. Firstly, BioLit (http://biolit.ucsd.edu) attempts to bridge the database and journal worlds [6]. Databases are rich in semantics, which are most often manifest in the form of a database schema with associated referential integrity to strictly impose access to those semantics. On the other hand, journal text, as we have seen above, is generally bereft of controlled access to those semantics. Nevertheless, the results of natural language processing and unique terms like database identifiers found in full journal text can be used to extract some semantic meaning and impose useful markup. This opens up the possibility of integrating database and literature content, which is one goal of BioLit, using the PLoS corpus and the RCSB Protein Data Bank (PDB; http://www.pdb.org) as a test bed. Of course, the best way to introduce semantic markup into a journal article is to capture it at the time the article is written. To do this is another goal of the BioLit project, in collaboration with Microsoft. In the same way that a spellchecker compares every word of a written article, suggesting changes as needed, a semantic checker can use existing ontologies and pseudonym tables to suggest formal definitions and subsequent tagging of semantically relevant content for a variety of uses, for example, integration with database content and more directed searching. Open access literature provides a rich dataset to experiment with these ideas.

PubNet visualizes relationships based on the results of a PubMed query (http://pubnet.gersteinlab.org) [7]. Using a standard PubMed style query, articles can be retrieved and associations developed by further retrievals. Associations are presented as graphs where nodes represent the terms and edges represent the relationship between them. A favorite query is to construct your own publication net that shows all your co-authors and how they have published with you and each other. A more generic example can be found in a recent article that showed the emergence of the RNAi field and the interrelationship of authors publishing related work in this field [8]. Associations can be made between data items such as PDB identifiers, UniProt identifiers, and the like. PubNet operates on PubMed XML output, which includes only the publication details and abstract of the paper, so it is not taking advantage of the full text of the paper. However, it could be readily expanded to do so if the rest of the paper were included in the XML output. It is easy to imagine how connections between results and specific entities (like protein identifiers) across a large body of literature can begin to yield interesting and provocative relationships.

SciVee (http://www.scivee.tv) [6] caters to the YouTube generation of video consumers; after all, they are the next Nobel Laureates. Using PLoS and other content taken from PubMed Central, SciVee provides a video-on-demand service that mashes up video provided by the authors and the paper content into what is called a “pubcast.” As the growing body of scientific literature threatens to overwhelm us, we are faced with either an abstract, which is consumed in a minute or two, or a full paper, which may take two to three hours to absorb in detail. SciVee's notion is that an intermediate amount of content is needed. Who better to provide this intermediate view than one of the authors by giving a five-to-ten-minute video presentation of the content of the paper? If only the abstract of the paper is available, the story ends there—a video and abstract side by side. If the full text of an open access article is available, additional possibilities emerge. The paper may be synchronized with the video, so as the author talks, appropriate tables, figures, and text are brought into view (see http://www.scivee.tv/node/5275 for an example). Alternatively, upon a single click, the author may pop up and explain a particular segment in more detail. Authors of accepted PLoS papers are invited to make video segments and upload them to the SciVee Web site. This can be done using a webcam and software standard on a PC or Mac, or done more professionally. Our experience has been that they cost about US$150 at our home institutions using one of the available media services—just a natural evolution from the days when we used to make 35 mm slides for a presentation. However, unlike slides which were viewed a few times by a select audience, pubcasts are viewable by a worldwide audience at any time. We do know already that the availability of online synchronized open access content generates interest in the online version of a paper, perhaps bringing a new audience to the work, and it remains to be seen how it improves the comprehension and learning experience.

Podcasts may be what the reader is seeking when video seems like overkill. Audio tracks could be associated with major figures or other visual elements taken from the open access paper. Perhaps a podcast of the traditional journal issue is desirable: while jogging or walking to the laboratory you could get an overview of the latest issue of this journal, presented either by the authors of papers in that issue or by a journal editor. This takes eToCs to a new level and medium. It seems that every student walking around campus has the means in their hands and ears to take advantage of this today. This could also benefit scientists with disabilities. *Science*, *Nature*, and other journals are using podcasts regularly, and they seem to be well received.

Certainly open access journals, such as the PLoS journals, have an opportunity to try and develop those killer apps. PLoS is using the TOPAZ application framework for a publication application built on a semantic repository. TOPAZ allows users to add notes directly to the article content and to add comments to the article. The published article then becomes the basis for an evolving discussion within the scientific community rather than a static article. The user notes are also stored as relationships to the article which can be later mined to uncover new connections in the research. The journals *PLoS ONE* and *PLoS Neglected Tropical Diseases* are published using the TOPAZ application framework, and other PLoS journals have just started using the same framework.

Another long-term notion at PLoS is that of portals or hubs in which selected materials from across the journals (and from open access literature as a whole) can be brought together by readers to form their own personalized view of the literature, or by special interest groups to share with emerging communities.

Let us consider some other opportunities, hopefully to whet your appetite for creating your own killer apps. So far, open access publications have been viewed by their readership (and often by their publishers) in very traditional ways. That may be changing; consider the ability to comment on a paper. This journal now offers readers the ability to comment on any aspect of a published paper for all to read, and we certainly invite you to comment on this Editorial. Many of you may not think twice in sending a comment to a list server or blog; however, you may perceive that as a different medium with a different social or professional context, and it may provide anonymity.

Perhaps a video about a paper as described above can also overcome the stigma about rating a paper itself? Certainly rating a paper would seem reasonable when done by the Faculty of 1000 (http://www.f1000biology.com), but it is not a generally accepted practice. We challenge you to rate this Editorial too. In some ways the reluctance to rate a scientific paper is strange since we suspect the same person may well rate a book on amazon.com. Another option would be to add a Digg or del.icio.us button (http://digg.com or http://del.icio.us) to incorporate conventional media ranking tools into an academic journal Web site. If one finds an interesting article, one could immediately flag it with these tools. *The New York Times*, *PNAS*, and many other publications already offer this possibility, which would be an interesting vehicle for us authors and readers, both to get quick user feedback on interesting articles and to leverage mainstream tools. Taking this a step further is to introduce the idea of folksonomy, where readers themselves tag the articles with semantically useful (and hopefully) controlled terms as a way to provide semantic content. In the life sciences this is simply an extension of what annotators at the National Library of Medicine do in associating Medical Subject Headings (MeSH) to papers, including those in this journal. The difference proposed here is that the content is controlled by the community of readers.

A related concept, which has been nominally explored by *Nature* [9] and others, is giving the referee the option to make his or her review public. In addition to communicating comments exclusively to the editor and to the authors (usually anonymously), one could also elect to have one's referee report, or parts of it, made public on the Web with the published article, either in a personalized or in an anonymous fashion. This would generate an incentive for referees, allowing them to get recognition for their work, as readers would see directly the referees' names and their comments associated with each article. We could allow authors to post their formal response to referees on the journal Web site as well. Referees and authors make tremendous efforts putting together reports and responses, and making them publicly available would be a way for the journal and the community as a whole to get some additional value from this content by providing direct commentary on the article's strengths and weaknesses and by giving didactic clues to students and post-docs. We feel open review has the possibility of improving the review process immensely, but also expect objections from some authors and reviewers.

These are a few ideas that we have come up with for making use of the wealth of knowledge contained in open access articles. We feel that it is now time for the community represented by this readership to act. What say you? It is important we hear from you on the subject of better use of open access content. At the forthcoming Intelligent Systems in Molecular Biology Conference there will be a session on Scientific Publishing where these views will be discussed, and we also encourage feedback via e-mail, blog, or article comment.
